# ‘The Health-Secure Partnership’: Study protocol for the development of a school- & community-based intervention for promoting healthy nutrition among rural adolescents in The Gambia

**DOI:** 10.1371/journal.pone.0327164

**Published:** 2025-07-17

**Authors:** Haddy Jallow-Badjan, Anne-Marie Bagnall, Tanefa A. Apekey, Haddy Tunkara Bah, Haddy Crookes, Ramatoulie E. Janha, Fatoumatta Kassama, Hassan Njie, Oritseweyinmi Erikowa-Orighoye, Thomas Senghore, Tayamika Zabula, Maria J. Maynard, on behalf of the Health Secure Partnership consortium

**Affiliations:** 1 Migrant Health Research Group, School of Health, Leeds Beckett University, Leeds, United Kingdom; 2 Centre for Health Promotion Research, Leeds Beckett University, Leeds, United Kingdom; 3 Sheffield Centre for Health and Related Research, University of Sheffield, Sheffield, United Kingdom; 4 School of Medicine and Allied Health Sciences, Department of Nursing and Reproductive Health, University of The Gambia, Banjul, The Gambia; 5 The National Nutrition Agency of The Gambia, Banjul, The Gambia; 6 Medical Research Council Unit The Gambia at the London School of Hygiene and Tropical Medicine, Banjul, The Gambia; 7 Girls Pride Gambia, Tallinding, Serrekunda East, The Gambia; 8 National Health Insurance Authority, Sukuta, The Gambia; 9 Arden University, Coventry, United Kingdom; 10 School of Medicine, University of Leeds, Leeds, United Kingdom; MRC Unit The Gambia at LSHTM, GAMBIA

## Abstract

**Introduction:**

Interventions addressing malnutrition (under- and overnutrition) among adolescents have not been developed in The Gambia. We aim to coproduce the first phase of *‘The Health-Secure Partnership’ (HSP)* – an innovative multicomponent intervention for adolescents in rural areas of The Gambia. Inference drawn from existing evidence and public contribution suggest that the future intervention would ideally involve nutrition-specific components coupled with changes to wider school and community contexts, delivered in partnerships between schools and local communities.

**Methods:**

The design of *HSP* is underpinned by health promoting school and community centred approaches, informed by a systems-thinking lens. The project activities will be conducted in three linked stages. Stage 1: Current nutrition-related activities and environments will be observed in four rural Gambian schools, their local village communities, and one additional village which does not have a school. Around 50 key stakeholders (such as teachers, parents, community members, and wider systems stakeholders) will complete questionnaires on their views on the current nutrition-related context and systems, cultural norms, and actions needed to address adolescent malnutrition. Focus groups and interviews will then be conducted with approximately 70 adolescent girls and boys to understand their experiences and opinions about their intervention needs. Stage 2: Potential intervention components, session content and mode/s of delivery will be explored in meetings and workshops with the consortium and stakeholders (including adolescents) to achieve consensus on the intervention format. Examples of session content, and intervention resources will be made, and a community event held to see what the public think of the ideas and materials. Stage 3: A description of the intervention and a codesigned evaluation plan will be documented. Dissemination will include publications, presentations, song and drama to suit a range of audiences (adolescents and their communities, other stakeholders, academics, and policy makers).

**Discussion:**

This study protocol presents our plans for the *HSP* intervention development project, addressing a critical gap in the evidence base. Findings will inform subsequent research phases exploring the feasibility and acceptability of the intervention among adolescents.

## Introduction

Adolescence is defined by the World Health Organisation as the phase of life from age 10–19 years [1]. As the period between childhood and adulthood, it is a unique stage of human development and an important time for laying the foundations of current and later good health, among contemporary and future generations [2]. Both undernutrition [underweight, stunting, and/or micronutrient deficiency] and overnutrition [overweight and obesity] impact significantly on health during adolescence and across the lifespan [1].

In 2023 24% of the population in The Gambia were adolescents [3]. Available age-specific national data indicate that 28% of 15–19-year-olds in The Gambia are underweight, 12% are overweight or obese, and 43% have anaemia [4]. Previous studies in rural Gambia, including research led by the authors [5–9], provides intelligence on what shapes adolescent nutrition. Recent quantitative studies fill knowledge gaps about adolescent nutritional status [5], and the individual and wider drivers of adolescent diet, physical activity (PA) and malnutrition [6,7]. Routine childhood anthropometry data show that, despite a significant decline in childhood undernutrition, rates remain high in rural Gambia [9]. Qualitative studies in The Gambia provide rural adolescents’ own insights into knowledge, attitudes and beliefs, community contexts, and cultural norms relating to diet, PA and weight [7,8]. Understanding gained of adolescent girls’ views on research approaches (engaging communities, optimal recruitment methods, prevention and treatment of under- and overweight) can also be applied to intervention development [7]. Current knowledge is yet to be harnessed in coproduced actions to improve the nutritional status of Gambian adolescents. Although adolescent nutritional health is integral to the existing policy base [10,11], it remains a neglected area of these strategies. Half of the billion adolescents globally live in sub-Saharan Africa [3]. Identifying affordable solutions in The Gambia, with attention to systems, will inform applicability of low cost actions to address adolescent malnutrition for other sub-Saharan African countries.

Overall, attendance at secondary school in The Gambia is high (87%) [11] and in rural areas, due to late starting age at primary school, pupils are regularly in secondary school until they are 19 or 20 years old [6]. This makes schools in The Gambia, as elsewhere, an obvious place to conduct nutrition-related intervention activities, which in turn are likely to improve education outcomes due to enhanced cognitive performance [12]. There is strong evidence that school based nutrition interventions(SBNIs) improve cognitive ability, nutrition knowledge and micronutrient status of schoolchildren in sub-Saharan African countries [13]; and some evidence of the potential to improve anthropometric outcomes [13]. We posit that a systems-informed multicomponent approach to developing and evaluating complex interventions [14] will improve the future efficacy of SBNIs. Further, gender equity in access to primary [elementary] school in The Gambia decreases at secondary [middle/ high] school, falling to a ratio of 1:2 females to males enrolled in school [3]. Girls in The Gambia are also more vulnerable to under- and overnutrition than boys [15,16]. A novel approach which is sensitive to context and involves families and communities will enhance the potential for gender-responsive actions, and thus gender-equity in intervention effectiveness. We will conduct theoretically underpinned and methodologically rigorous intervention development research, incorporating learning from our ongoing intervention development work [17], adapted to the current setting. The overall aim of the project is to develop ‘The Health Secure Partnership’ (*HSP*). *HSP* is an innovative, new multicomponent school- and community-based intervention which tackles undernutrition and prevents further rise in overnutrition among adolescents in rural areas of The Gambia. Intervention components will be codeveloped within the project, prioritising potential beneficiaries’ views; however existing evidence, public contribution and theoretical approach suggests certain features are needed. The intervention is thus likely to include nutrition-specific components (e.g., food supplementation; interactive nutrition education; tailored PA sessions). Additionally, inclusion of nutrition-sensitive components resulting in changes to wider school and community contexts (e.g., school gardening; improving water, sanitation and hygiene [WASH] facilities; teacher and community health worker training) is indicated. Addition of actions that change the system’s goals (i.e., the system’s underlying paradigm), such as maintaining stakeholder fora as part of the intervention, will be consistent with our systems lens [18]. Delivery of the intervention is anticipated to be via partnerships between schools, local communities, and supportive system level infrastructure.

The project aim will be addressed through six objectives in three linked stages. Stage 1, coproduction planning: (1) Conduct consortium consolidation activities; (2.1) Observe and map the current nutrition-related context and systems in four rural Gambian schools and five local communities (one without a school); and ask around 50 key people (such as parents and community health workers) to complete questionnaires on their views on these topics; (2.2) Conduct focus groups and interviews with approximately 70 adolescent girls and boys to understand their opinions about their intervention needs; (3) Gather examples of existing intervention resources and local practice. Stage 2, codesigning and creating: (4) Generate ideas about intervention features; (5) Decide on potential intervention sessions, their content, and how they will be delivered, make examples of session content (e.g., game-based learning; cooking session plans), and hold a community event to see what the public think of the ideas and materials. Stage 3, documentation and evaluation planning: (6) Document an intervention description and a codesigned evaluation plan.

## Methods

A mixed methods study design will be employed. The project started in August 2024 and will take place over 18 months, ending in February 2026. We are a diverse consortium with academic members from universities in the United Kingdom and The Gambia. Non- academic members are in The Gambia and include the National Nutrition Agency (NaNA), public contributors (adolescents; parents; village development committee (VDC) representatives, educators and community health workers), a third sector youth organisation lead, and senior health and education policy makers.

### Project setting

The majority of the coproduction and data collection activities will be conducted in Mansakonko Local Government Area (LGA), one of the eight LGAs in The Gambia. Mansakonko is chosen due to its make-up of predominantly rural villages (80%); the large number of adolescents (n = 20,521) in these rural areas; the levels of poverty and limited infrastructure (road networks, electricity, health facilities) resulting in these youth (and the wider population) being highly vulnerable to risk of malnutrition [4]; and its accessibility (avoiding crossing or circumventing the river Gambia). The population is predominantly of Mandinka ethnicity (80%) with the remaining population of Fula (16%), Jola (3%), and other (1%) ethnic groups [19]. English and Mandinka languages are spoken across groups. Schools are taught in English or Arabic (these schools are known as Madrassas). Relevant schools in the region include both English-taught institutions and Madrassas. As a predominantly Muslim society, children of this faith go to both types of school, but those of the minority Christian faith only attend English taught schools. School attending adolescents will be included via a selection of these schools, and adolescents not attending school via local communities (see Stage 1, below). This setting is an area of significant need [5,9] and will provide a case-study for wider implementation and sustainability of the programme in other rural areas of The Gambia.

All procedures will be conducted in accordance with the Declaration of Helsinki. Ethical approval has been obtained from the Research Ethics Committee, School of Health, Leeds Beckett University (Reference: 136815), The Gambia Government/MRC Unit The Gambia at LSHTM Joint Ethics Committee (Reference: 31344), and the Regional Education Directorate 4, Mansakonko, The Gambia. Permission will also be sought from participating village Chiefs in Mansakonko. Participation in the research will be voluntary and to ensure informed consent respondents will receive a participant information sheet (PIS) about the study, prior to deciding to take part. Information will be provided in varied formats: written, spoken, and spoken in Mandinka (the predominant language in the setting) to accommodate diverse literacy needs. Consent will be certified with a signed consent form (parents; other adults, and adolescents aged 18 or 19 years) and assent form (children aged under 18 years). However, in line with supporting those with low literacy, verbal consent can be obtained in the presence of and countersigned by a literate, impartial witness (e.g., literate family members, friends or colleagues of the prospective participants). Focus groups and interviews will be recorded with permission, and as certified on the consent form. Study participants will receive a thank-you gift at a level and nature (e.g., school book/ stationery) that is not likely to coerce subjects into taking part but will demonstrate the research team’s acknowledgement of participants’ time and contribution of their views. All data will be stored ethically and securely in accordance with the host institution data protection policy and in line with the UK General Data Protection Regulation [20]. This is described in the data management plan incorporated within the funding and approved ethics applications.

### Development approach and underpinning theory

The project is shaped by the Medical Research Council (MRC) framework for complex interventions [14]. Employing a development framework is recommended by the MRC guidance [21]. A combined ‘partnership’, and ‘evidence and theory’ approach [22;p.6], will be our intervention development framework. Partnership will take the form of a participatory coproduction model: all subgroups of the consortium will work together from the outset, consistent with systems-informed intervention approaches [23]. The development process is underpinned by health promoting school and community centred approaches, behaviour change theory and a systems lens.

To effectively foster health promotion, schools need the support of wider community and societal actors [24]. This holistic ethos for improving the health status of school aged children gave rise to the World Health Organization (WHO) Health Promoting School (HPS) concept which informs the current project. A health promoting school is characterised as constantly strengthening its capacity as a healthy setting [25]. However, the techniques used to engage families in school-based interventions have been deemed inadequate and unlikely to lead to significant behavioural change [24]. Our work is therefore also informed by the ‘family of community-centred approaches for health and wellbeing’ [26;p.356]. These approaches do not merely target populations to receive activities; rather, they seek to mobilise community assets, promote health and wellbeing in local settings, and work in partnership with communities to promote equity and peoples’ control over their health [26]. A systems-based approach to developing and evaluating complex interventions is recommended in The MRC framework. As such, we can view schools as complex adaptive systems [14], and that the multiple levels which may influence adolescent malnutrition (such as family, community, policy) are also recognised as systems with linkages, relationships, feedback loops [whereby one change reinforces, promotes, balances or diminishes another], interactions among the system parts, and dynamics between levels as core properties [14]. We will integrate the behaviour change wheel (BCW) to conceptualise individual-level change, with the associated capability, opportunity, and motivation model for influences on behaviour (COM-B) [27] within the underpinning theory. A systems-informed approach builds on the socio-ecological perspective we have used in previous intervention development work [17] and research in The Gambia [6,7].

In order to operationalise a systems informed approach across all stages of a research project, we will integrate the recently developed protocol for systems thinking across research (STAR) framework [problem description; design and planning and data collection/analysis phases] [18;p.4] into guidelines for development actions [22,28]. This will ensure that although targeting behaviours and structures, we will also identify the systems deepest beliefs (known as the system’s paradigm), system-level outcomes and perspectives [18].

### Activities and planned outputs

The project activities will be delivered in three interlinked stages, informed by the aforementioned guidance on intervention development actions [22, 28;p.7], and the STAR protocol [18].

### Stage 1: coproduction planning, objectives 1–3

#### Consortium consolidation.

A kick-off meeting was held in October 2024 with subsequent bi-monthly hybrid in person meetings to review the project progress. Two face- to- face consortium workshops will be conducted in the rural setting. The initial workshop will provide the opportunity for the consortium to consolidate their shared values, vision, and principles, and confirm the study protocol. The second workshop will be conducted during Stage 2 of the project (codesigning and creating, below). Coproduction will also be fostered/ supported by creative activities (gaming, drama) with the adolescent PIR group (3x meetings), and discussions with the adult PIR group members (e.g. VDCs; 3x meetings). This approach will ensure that coproduction activities centre and empower adolescents, and are inclusive with different ways for members to contribute to the project.

#### Mapping context and the wider system.

We will map the health promoting contexts in four rural schools and their local village communities, plus an additional village which does not have a school. The system mapping methods contribute to identifying the system’s boundaries, ‘boundary critique’ [exploring the different possible boundaries] [29;p.467], and the systems paradigm. Data will be collected through use of observation to describe and document environments and behaviours relevant to nutritional health. Short questionnaires will also be administered to a purposively selected, convenience sample of key informants in the school and community settings. We will focus on beliefs, values and practices of teachers, parents, community health workers, and other community members; and factors in the context perceived to be potential barriers for the process of change. Context mapping will be completed by combining these data with policy and other literature review.

#### Understanding experiences, perspectives and needs of the target populations.

Semi structured focus group discussions (FGDs) will be conducted with adolescents to understand their experiences, perspectives, and intervention needs, adding to existing intelligence outlined above. The key benefits of FGDs are time efficiency and access to group norms and language through group interactions [30]. However, potential challenges with FGDs such as dominating or reticent participants, and overtalking among speakers require skilled handling to avoid negative impact on data quality [30]. These issues will be managed by the lead author (HJB) and fieldworkers (FWs), with appropriate skills in facilitating and supporting FGD, respectively (see data collection, below).

#### Sampling and sample size.

Mansakonko LGA has six districts: Kiang Central, Kiang East, Kiang West, Jarra Central, Jarra East and Jarra West. Four of the six districts (Kiang West, Kiang Central, Jarra East and Jarra Central) in the region were randomly selected using randomly generated numbers in MS Excel. A total of five rural villages within the chosen districts will also be randomly selected, within pragmatic groups based on the types of schools present. Thus, two of all of the villages with English-taught schools, and two from all of the villages that have madrassas will be selected. Within the district with the highest population density, the fifth village will be randomly selected from those which do not have a school, and whose adolescents are attending a school in a different village in that district that has been recruited to the study. Within the four selected villages with schools, a total of four schools will be invited, again taking a combined random selection and pragmatic approach (i.e., to ensure representation of the different school types, early [aged 10–15 years] and late [aged 16–19 years] adolescence). The four village communities in which the schools are placed will be included in the context mapping, plus the additional village chosen because it does not have a school.

We will aim for at least 10 participants in each of the key informant groups, and allow for an additional 10 relevant system stakeholders identified through snowball sampling (~n = 50 in total). This sample size will permit stakeholder-subgroup analysis without risking re-identifying individuals in anonymised data.

Focus group participants will be purposively sampled with regard to age, gender, ethnicity, religion and socio-economic circumstances. Together with the choices of villages and schools (above), this will ensure inclusion of adolescents with diverse characteristics and thus a range of voices in the sample [31]. Eight FGD, with 6–8 adolescent participants per group, will be conducted across the four participating schools, two in each school. This will allow for age and gender homogenous FGD (females aged 10–15 and 16–19 years; males aged 10–15 and 16–19 years). FGDs will also be complemented by a small number of one-to- one interviews (~n = 6), focusing on the age-groups/ genders found to be not regularly attending school. Thus, the target sample size is approximately 48–70 adolescents.

By design, the aim of the sampling strategy and target sample size was to support obtaining rich, exploratory accounts and not statistically representative data.

#### Recruitment.

Project awareness raising will commence in February 2025, and subsequently recruitment will be conducted from April to June 2025, and continue iteratively until purposive sample targets are achieved. We have found in our previous work that word- of- mouth awareness raising via focal link persons to be the most effective recruitment method [6,7,15,16]. Identified through consortium members’ networks, there will be link personnel to connect the researchers with each school, and community leaders such as the VDC representatives will link researchers with the communities. Liaison with the school link personnel and community leaders will facilitate recruitment of the purposive, convenience sample for the key informant questionnaires, and snowball sampling of new participants referred by study respondents. School attending adolescents will be recruited within the four included schools facilitated by the school link personnel, and adolescents not attending school will be recruited via the participating local communities, aided by the community focal persons.

#### Data collection.

Data collection will take place alongside recruitment from April to June 2025. Data collection will be conducted by HJB, a Gambian post-doctoral researcher with over 10 years’ experience as a researcher (with qualitative focus group and interview facilitation skills) and as a health professional working with the target populations. HJB will be supported by two fieldworkers, experienced in supporting FGDs (ice-breaker exercises; notetaking). HJB and the FWs are multilingual, including fluency in spoken and written Mandinka; therefore, participants will be able to use their preferred language (English or Mandinka). Observations will be aided by an observation checklist informed by key literature exploring food and physical activity environments in African settings [32,33], and WASH assessment [34]. Key informants will complete an anonymous questionnaire in the school and community settings. Questionnaire design was informed by literature around soft complex systems mapping approaches, and factors that influence the rate and success of innovations such as health interventions [35,36].

The FGDs will be guided by a topic guide based on the goals of the project, our prior work and other literature, and input from the consortium including adolescents. Topics include the school and community environment, food and activity beliefs, values and practices, nutrition and activity engagement and knowledge, potential barriers and facilitators of healthy eating and active living, and views on ideal intervention content and delivery. The FGDs will be conducted in the schools, and the interviews in the relevant village compounds, at times that do not interfere with studies or other important activities. Participant characteristics (age, gender, ethnicity, religion, socio-economic indicators) will be captured via a short questionnaire, with completion supported by HJB and the fieldworkers. Creative methods will be used (as for the coproduction sessions) as ice breakers and to aid eliciting participants’ stories [30].

All the materials that will be used to inform this stage (PIS, consent form, semi-structured topic guide, sociodemographic questionnaire) were coproduced within the consortium. Specific group discussions were held with the public contributors to ensure their views on the materials were included.

#### Data analysis.

Checklist and questionnaire data will be entered into Excel spreadsheets for data management prior to descriptive statistical and content analysis (counts and percentages of individuals or themes) in SPSS v.29. FGD/ interview recordings and field notes will be translated/ transcribed verbatim to facilitate inductive thematic analysis, informed by the underpinning theory [37]. Reading and re-reading transcripts will aid familiarisation with the data. Subsequent analytical stages will include conducting initial coding, generating preliminary themes, reviewing, developing and refining themes, and reporting on the findings. The target sample size is likely to be sufficient to reach data saturation [38], although not guaranteed. NVivo R1 software will be used to support coding consistency. Iterative data collection and analysis, and charting the anonymised participants’ data excerpts in Microsoft Excel will aid exploring data saturation and verifying the plausibility of themes identified.

*Output:* a summary report of the mapping and qualitative findings to support codevelopment of intervention goals and programme theory

#### Codevelopment of intervention goals and programme theory.

*Data synthesis:* Data will be synthesised in narrative summaries focusing on intervention needs of adolescents, and the strengths and limitations of behaviour change approaches relevant to stated needs. To aid understanding for non-academics, visual summaries (including videos, which the team has experience in making accessible to those in low resource setting) will be produced by the public contributors, supported by the researchers and other collaborators. The data sources include: (1) data from objective 2 (mapping, focus group and interview analysis); (2) existing literature, including our existing compendium of effective evidence-based behaviour techniques, community engagement and health promotion guidelines [17]; and (3) available intervention components and other resources that can potentially be adopted/ adapted (with permission), in addition to developing our own novel components and resources.

#### Theory mapping.

The narrative summaries will be shared with the consortium for review and feedback and final adjustments will be made in the bi-monthly/ public involvement group meetings. Findings will be mapped to and develop the current theoretical framework.

*Output:* A systems map and logic model of change, system levers and influencing factors.

### Stage 2: codesigning and creating, objectives 4 & 5

#### Generate ideas about intervention features.

The core team will generate ideas about intervention components and features using the logic model to produce a theorised list of the components, their content and delivery, consistent with the underpinning theory.

*Output:* A theorised list of potential components, content, and mode/s of delivery.

#### Make decisions on the intervention content, format and delivery.

We will achieve consensus on intervention content, format and delivery, process and outcome evaluation items and methods for the future research phases at the second coproduction workshop and ancillary meetings for specialist input (e.g., from the health economists). Examples of session content (e.g., game-based learning; cooking session plans) will be made by the core team. A one day community dissemination event will also be organised in one of the participating villages, with approximately 100 participants including potential intervention beneficiaries, other community members, the consortium team, and additional relevant stakeholders from the wider system. Interim findings will be shared, including via *‘Keneleng’* (informal women’s singing and drama groups). Attendees will be encouraged to share their views on the plans for content, example materials, format and delivery of the intervention which will be incorporated into the documentation stage.

*Output:* Intervention content, format, and delivery decisions; prototype materials; a formal implementation plan.

### Stage 3: documentation and evaluation planning, objective 6

#### Document an intervention description.

We will document and disseminate an intervention manual detailing its content, format and proposed delivery models, aligned with the template for intervention description and replication (TIDieR) checklist and guide [39,40]. An evaluation plan will be codesigned with stakeholders. This will detail the evaluation design, objectives of future process and outcome evaluations and associated measures, and consideration for participant recruitment [22], contributing to the next stages of funding acquisition. Results from the project will be generated incrementally from June 2025 to February 2026. These findings, incorporating the outputs listed in preceding stages (e.g., the systems map and logic model of change), will be reported for academic audiences in publications and presentations, informed by guidance for reporting intervention development studies in health research (GUIDED) [41]. Outputs suitable for diverse audiences, particularly adolescents and their communities, will include short films (including those featuring the feedback shared by the *Keneleng* singers) and an infographic. Collectively, these outputs are the key benefits from this project. The transparent research process, careful documentation and varied dissemination of outputs will maximise the impact of the project outcomes. Predicted outcomes are that we will add new knowledge to the evidence base for what is likely to be effective in addressing malnutrition among adolescents in The Gambia; and that the outputs will be used to refine and test the intervention.

*Output:* An intervention manual and a codesigned evaluation plan.

### Public involvement in the research

The views of our existing public contributors (adolescents, VDC representatives), and people that work closely with them (e.g., community health workers; NGOs) [6] have been included in the project design. These views signal readiness for intervention that is sensitive to cultural practices. Aligned with existing qualitative research, the need to address the various levels of influence on adolescent nutrition was voiced. We are mindful of concerns about research ultimately being for the benefit of adolescents and their communities. This wider group was drawn on to form a project specific public involvement group of 4–6 adolescents, two parents and teachers and two community leaders. This group’s contribution will be embedded in the intervention development process, as summarised in the methodology.

## Discussion

An affordable and gender-inclusive intervention to address adolescent malnutrition does not currently exist in The Gambia. To date, adolescent health action in The Gambia has largely focused on infectious disease prevention and management [42]. The design of context specific, theory-informed interventions to address adolescent nutritional health in The Gambia, is timely. Adolescents are explicitly mentioned in 12 health-related Sustainable Development Goal indicators including nutrition [10]. The United Nations Global Strategy for Women’s, Children’s, and Adolescents’ Health [43] estimates high returns from investment in evidence-based interventions. The Global Accelerated Action for the Health of Adolescents [44] urges evaluation of participatory action strategies with adolescents as highly cost-effective in improving adolescent health and reducing future adult burden of disease. We therefore propose developing an innovative multicomponent intervention likely to include nutrition-specific components and changes to wider school and community contexts, delivered in partnerships between schools and local communities. This will address a critical gap in the evidence base. Progression to the next phase will be indicated by success in producing outputs from this current development phase (coproduced systems map and logic model of change; an intervention manual describing the intervention structure, components, and delivery; and example intervention resources). Assuming success, the next phase will include feasibility and acceptability testing, and intervention refinement, which may subsequently be linked to a pilot cluster randomised trial. As is the case for the next phases of our previous intervention development work [17], mixed-methods process evaluation, intermediate outcomes, and cost-effectiveness evaluation will be planned. This will aid insight into success, failure, and/ or unexpected consequences [14]. Furthermore, to expand on this strategy, diverse evaluation methods will be used to ensure that, as well as quantifiable elements, systems concepts: *emergence* [emergent properties that are features of the system as a whole]; *feedback*; and *adaptation* [change of system behaviour in response to an intervention] [14] are incorporated. Evidence of feasibility and effectiveness will lead to further refinement, optimisation of intervention efficiency, an implementation, upscaling, and sustainability strategy. We will review emergent *‘wholistic’* approaches integrating implementation and systems frameworks [45;p.12] in planning future research phases.

The consortium is dedicated to the coproduction approach. Members have worked closely with adolescents and others in Mansakonko, building trust, and we have ongoing public contribution from adolescents and influential community leaders. Additional support for public contributors will ensure their vital input is encouraged and empowerment is fostered. The project’s research, policy and practice partners and collaborators, with longstanding presence in the chosen setting and links with communities, ensures familiarity with research among potential participants. Experience on the ground has been important in the project planning, e.g., knowing best time periods for data collection. Little knowledge of systems-informed approach for some consortium members is challenging. However time allocated to presentations and discussions in consortium meetings, sharing the expertise that is within the team, will foster knowledge development across the group.

## Conclusion

Intervention during adolescence has the potential of improving current health, the social and economic development, and the health of future generations. We aim to develop a new multicomponent intervention for adolescents in rural areas of The Gambia, to be delivered in partnerships between schools and local communities. Multicomponent interventions, codeveloped with a systems lens, represents a step-change in efforts to address malnutrition among adolescents in The Gambia. This work will also contribute to evidence of how complex systems approaches can be operationalised to address public health problems in low resource settings. Comprehensive documentation of the development process will increase the chances that the intervention has wide applicability in real world conditions beyond the research project.

## Supporting information

S1 Appendix Protocol submitted to ethics committees. (PDF)
