# Supplementary material for: ‘The Health-Secure Partnership’: Study protocol for the development of a school- & community-based intervention for promoting healthy nutrition among rural adolescents in The Gambia
Source: PLoS One. 2025 Jul 17;20(7):e0327164. doi: 10.1371/journal.pone.0327164 (PMC12270090; doi:10.1371/journal.pone.0327164)
Supplement: S1 Appendix — (PDF) [file pone.0327164.s001.pdf]

STUDY PROTOCOL

The ‘Health-secure partnership’: a school- & community- based intervention for promoting healthy nutrition among rural adolescents in The Gambia

Study Acronym: HSP

Version 1 12/9/24

SPONSOR: Leeds Beckett University

FUNDERS: Medical Research Council: APP30152

Contents

1. Project aim and overview 3
   1.1 Study objectives 3
   1.2 Study team 3
   1.3 Background 3
   1.4 Project setting 4
   1.5 Intervention development approach 4
   1.6 Underpinning theory and systems lens 4
   1.7 Public involvement and collective input to the proposal 5
2. Methodology 6
   2.1 Stage 1 Coproduction planning (Months 0-6): 6
   2.2 Stage 2: Co-designing and creating (Months 7-9): 8
   2.3 Stage 3: Documentation, evaluation planning and dissemination (Months 10-13): 9
3. Future development phase 12
   3.1 Type of intervention, form and function 12
   3.2 Programme theory 12
   3.3 Intervention development and pathway to the next stage 12
4. Team capability 13
5. Data management and sharing 15
6. Ethics and responsible research and innovation (RRI) 18
7. Research involving human participation 19
8. References 20
9. Example Appendices 23

1. Project aim and overview

The overall aim of the project is to develop ***'The Health Secure Partnership'*** – an ambitious, new multicomponent school- and community- based intervention which tackles undernutrition and prevents further rise in overnutrition among adolescents in rural areas of The Gambia.

1.1 Study objectives

Underpinned by a systems perspective, behaviour change theory, and coproduction, the six objectives in three linked stages are to:

Stage 1, objectives 1-3

1. Conduct consortium consolidation activities (the consortium is comprised of researchers, project partner The National Nutrition Agency of The Gambia [NaNA], and a public involvement in research (PIR) group).

2.1. Observe and map the current nutrition-related context and systems in four rural Gambian schools and their local communities; and ask around 50 key people (such as parents and community health workers) to complete questionnaires on their views on these topics.

2.2. Conduct focus groups and interviews with approximately 70 adolescent girls and boys to understand their opinions about their intervention needs.

3. Gather examples of existing intervention resources and local practice

Stage 2, objectives 4 and 5:

4. Decide on potential intervention sessions, their content, and how they will be delivered. The project group will discuss the decisions in meetings and a workshop to agree on the best ideas.

5. Make examples of session content (e.g. game-based learning; cooking session plans) and hold a community event to see what the public think of the ideas and materials.

Stage 3, objective 6:

6. Write an intervention manual, and to share the project findings in ways that are suitable for different audiences.

1.2 Study team

We are a diverse team ('the consortium'), of researchers [from Leeds Beckett University (LBU; lead organisation), other UK universities, University of The Gambia (UTG); MRC Unit The Gambia at London School of Hygiene and Tropical Medicine (LSHTM)]; project partner NaNA, responsible for coordinating nutrition related activities in The Gambia; public involvement in research (PIR) group in The Gambia [adolescents; parents; village development committee (VDC) representatives; educators and community health workers], supported by a third sector organisation lead; and senior policy contributors from Gambian health and education government departments. Jointly the consortium will provide the infrastructure for the research to take place, ethical and inclusive research design and delivery that engages with relevant stakeholders.

1.3 Background

Existing research elucidates the co-existence of under- and overnutrition, and the impact of a range of determinants on unfavourable PA and dietary practices among adolescents in The Gambia [1-3]. Previous work of the team and others reveals nuanced understanding of how adolescents themselves perceive the interactions between structure (e.g. food availability; access to supplementation programmes) and cultural practices (e.g. food and PA-related gendered norms; aversion to taking supplements in tablet form) [2,4]. We have begun to understand adolescents' views on acceptable intervention actions (e.g. gender-specific sports activities; school-gardening [2,4]), and expectations of wider societal contribution to their

nutritional wellbeing [5]. Further, adolescent girls' views on optimal recruitment methods, feasibility and acceptability of data collection tools (such as anthropometric and biological measures) [5] inform the design and evaluation of interventions. We need to deepen our understanding of rural Gambian adolescents' perspectives on context, experiences and intervention needs, coproducing a programme with these young people at the centre.

1.4 Project setting

The majority of the coproduction and data collection activities will be conducted in Mansakonko Local Government Area (LGA), one of the eight LGAs in The Gambia. Mansakonko is chosen due to its make-up of predominantly rural villages (80%); the large number of adolescents (*n*=16,850) in these rural areas; the levels of poverty and limited infrastructure (road networks, electricity, health facilities) resulting in these youth (and the wider population) being highly vulnerable to risk of malnutrition [6]; and its accessibility (avoiding crossing or circumventing the river Gambia). The population is predominantly of Mandinka ethnicity (80%) with the remaining population of Fula (16%), Jola (3%), and other (1%) ethnic groups [7]. English and Mandinka language are spoken across groups. Schools are English taught or Arabic (known as madrassas). The schools in the LGA relevant to the target age-groups are senior secondary schools (SSS; adolescents aged 16+years) – English taught (x1) and madrassas (x4); English taught early child development (ECD) to grade nine schools (aged up to 15yrs; x17); and grade 4-9 madrassas (aged up to 15yrs; x14) [personal communication: Regional Education Director, policy consortium member]. As a predominantly Muslim society, children of this faith go to both types of school, but those of the minority Christian faith only attend English taught schools. School attending adolescents will be included via a selection of these schools, and adolescents not attending school via local communities (see objective 2ii). This setting is an area of significant need [1,8] and will provide a case-study for wider implementation and sustainability of the programme in other rural areas of The Gambia.

1.5 Intervention development approach

The project is shaped by the Medical Research Council (MRC) framework for complex interventions [9]. Employing a development framework is recommended by the MRC guidance [10]. A combined 'partnership', and 'evidence and theory' approach [11;p.6], will be our intervention development framework. Partnership will take the form of a participatory coproduction model: all subgroups of the consortium will work together from the outset, consistent with systems-informed intervention approaches [12]. The development approach is supported by the underpinning theories, outlined below.

1.6 Underpinning theory and systems lens

Health promoting school and community centred approaches:

Schools have an important influence on young people's health and wellbeing. To effectively foster health promotion, schools need the support of wider community and societal actors [13]. This holistic ethos for improving the health status of school aged children, gave rise to the WHO's Health Promoting School (HPS) concept which informs the current project. A health promoting school is characterised as constantly strengthening its capacity as a healthy setting [14]. However, the techniques used to engage families in school-based interventions have been deemed inadequate and unlikely to lead to significant behavioural change [13]. Our work is therefore also informed by the 'family of community-centred approaches for health and wellbeing' [15;p356]. These approaches do not merely target populations to receive activities; rather, they seek to mobilise community assets, promote health and wellbeing in local settings,

and work in partnership with communities to promote equity and peoples' control over their health [15].

It is recognised, however, that within a HPS ethos and community-centred approaches it is not only the setting that impacts on its actors (e.g. children, parents, community leaders) but also the actors that shape the setting [14]. A systems-based approach to developing and evaluating complex interventions is recommended in The MRC framework. As such, we can view schools as complex adaptive systems [9], and that the multiple levels which may influence adolescent malnutrition (such as family, community, policy) are also recognised as systems with linkages, relationships, feedback loops [whereby one change reinforces, promotes, balances or diminishes another], interactions among the system parts, and dynamics between levels as core properties [9]. A systems lens dictates that we build on our knowledge of this broader context, system stakeholders and system-level outcomes, how the intervention and system adapt to one another, and potential system-level influencing factors or levers [9,16], to successfully address complex problems such as adolescent malnutrition.

We recognise, as do others [17] that within complex systems, individual- or intrapersonal- level behaviour change, as well as population-level change, is required to improve population health. We will integrate the behaviour change wheel (BCW) to conceptualise individual-level change, with the associated capability, opportunity, and motivation model for influences on behaviour (COM-B) [18] within the underpinning theory.

A systems-informed approach builds on the socio-ecological perspective we have used in previous intervention development work [19] and research in The Gambia [5,20]. A systems lens gives greater focus to the context into which the intervention will, in the future, be introduced and intervenes directly on some of the structures within the system [21]. The combined application of these frameworks for addressing malnutrition is novel for The Gambia, and for low income sub-Saharan African countries more widely.

Operationalising systems principles across the project

A number of frameworks exist to guide the application of systems principles to public health research, but tend to focus on specific stages of the research cycle [22]. In order to operationalise a systems informed approach across all stages of a research project, we will integrate the recently developed protocol for systems thinking across research (STAR) framework [problem description; design and planning and data collection/analysis phases] [22;p4] into guidelines for development actions [11, 23]. This will ensure that although targeting behaviours and structures, we will also identify the systems deepest beliefs (known as the system's paradigm), system-level outcomes and perspectives [22, 24]. The STAR framework was informed by learning from the obesity prevention CO-CREATE project among adolescents in European countries and one LMIC country (South Africa) [25]. Exploring its applicability to addressing under- and overnutrition in The Gambia will extend understanding of the framework's value in low income African countries.

A preliminary systems map and logic model describes the development process, theory and intervention features (Figure 1).

1.7 Public involvement and collective input to the proposal

The views of our existing public contributors (adolescents, VDC representatives), and people that work closely with them (e.g. community health workers; NGOs) [20] have been included in this grant application. These views signal readiness for intervention that is sensitive to cultural practices. Aligned with the qualitative research presented, the need to address the

various levels of influence on adolescent nutrition was voiced. We are mindful of concerns about research ultimately being for the benefit of adolescents and their communities. We will draw on this wider group to form a project specific PIR group whose contribution will be embedded in the intervention development process, as summarised in the methodology. Views of the public have therefore fundamentally shaped the preparation of this proposal. Further, the proposal is the culmination of input from across the team.

Managing risks to delivery :*i. Lack of engagement of stakeholders and participants*. The consortium is dedicated to the coproduction approach. Members have worked closely with adolescents and others in Mansakonko, building trust, and we have ongoing public contribution from adolescents and influential community leaders (VDCs), as noted. The MRC Unit The Gambia at LSHTM Keneba rural field station has a longstanding presence in the chosen setting, and its links with communities ensures familiarity with research among potential participants. *ii. An ambitious research plan for the timeframe.* Consensus that the project can be delivered as planned was established in consortium meetings in preparing this bid. Experience on the ground has been important in the project planning e.g. knowing best time periods for data collection. The researcher co-lead (HJB) will lead the data collection supported by fieldworkers (FWs) from UTG, and is experienced in all of the data collection and coproduction methods. Previous work by HJB[9] demonstrates successful LBU-UTG joint working at pace. Learning from a previous MRC PHIND project nearing completion [Grant Ref: MR/X503022/1] [19], adapted to the current setting, also aids risk mitigation.

2. Methodology

The study aim will be achieved by six objectives within three interlinked stages, conducted over 13 months (also see Gantt chart, Figure 2). These stages are informed by guidance on intervention development actions [23,24;p7], and the STAR protocol [22].

2.1 Stage 1 Coproduction planning:

Objective 1. Consortium consolidation

Two face to face consortium workshops will be conducted in the rural setting. The initial workshop will provide the opportunity for the consortium to consolidate their shared values, vision, and principles, and confirm the study protocol. The second workshop will be conducted during Stage 2 of the project (co-designing and creating, below). The workshops will be full day events; meals and refreshments provided. Coproduction will also be fostered/ supported by creative activities (gaming, drama) with the adolescent PIR group (3x meetings), and discussions with the adult PIR group members (e.g. VDCs; 3x meetings). Bi-monthly hybrid in person/ video conference consortium meetings, and small meetings (e.g. one-to one meetings with policy actors in person or online, according to preference) will be held to review the project progress. This approach will ensure that coproduction activities centre and empower adolescents, and are inclusive with different ways for members to contribute to the project.

Objective 2i. Mapping context and the wider system

We will map the health promoting contexts in rural schools and their local communities. The system mapping methods contribute to identifying the system's boundaries, 'boundary critique' [exploring the different possible boundaries], and the systems paradigm [22]. A total of four schools will be invited taking a combined pragmatic (i.e. when there is one school to choose from) and random selection process (for choosing from several schools), to ensure representation of the different school types described above. The four village communities in which the schools are placed will be included in the mapping, plus an additional village without schools from which students travel to school. Data will be collected through use of observation

to describe and document environments and behaviours relevant to nutritional health. Short questionnaires will also be administered to a purposively selected, convenience sample of key informants in the school and community settings. We will focus on beliefs, values and practices of teachers, parents, community health workers, and other community members; and factors in the context perceived to be potential barriers for the process of change. We will aim for at least 10 participants in each of the four key informant groups, and allow for an additional 10 relevant system stakeholders identified through snowball sampling (~*n*=50 in total). This sample size will permit stakeholder-subgroup descriptive statistical and content analysis (counts and percentages of individuals or themes) without risking re-identifying individuals in anonymised data. Context mapping will be completed by combining these data with policy and other literature review (Objective 3i).

Objective 2ii. Understanding experiences, perspectives and needs of the target populations

Semi structured focus group discussions (FGDs) will be conducted with adolescents to understand their experiences, perspectives, and intervention needs, adding to existing intelligence outlined above. The key benefits of FGD are time efficiency and access to group norms and language through group interactions [26].

***Sampling and recruitment*.** FGD participants will be recruited from the schools and communities involved in the mapping exercise, in collaboration with the key informants and the education policy consortium member. The choice of schools and communities (above) and purposive sampling of individuals will ensure inclusion and a range of voices in the sample with regard to gender, ethnicity, religion and socio-economic circumstances. Eight FGD, with 6-8 participants per group, will be conducted across the four participating schools, two in each school. This will allow for age and gender homogenous FGD (females aged 10-15 and 16-19 years; males aged 10-15 and 16-19 years). FGDs will also be complemented by a small number of one-to- one interviews (~*n=6*), focusing on the age-groups/ genders found to be not regularly attending school. Thus the target sample size is approximately 48-70 adolescents.

***Data collection tools and procedures*.** A semi structured focus group schedule will be created based on the goals of the project, our prior work, other literature and input from the consortium, including adolescents. The FGDs will be conducted in the schools, and the interviews in the relevant village compounds, at times that do not interfere with studies or other important activities. If the one English taught SSS does not want to participate, we will pivot to conducting those focus groups in the community, of which we have prior experience[9]. HJB and the FWs are fluent in Mandinka, and the discussions will be conducted in the preferred language (English or Mandinka). Participant characteristics (as mentioned above) will be captured via a short questionnaire, with completion supported by HJB and the FWs.

Data analysis. FGD/ interview recordings and field notes will be translated/ transcribed verbatim to facilitate inductive thematic analysis, informed by the underpinning theory [27]. The target sample size is likely to be sufficient to reach data saturation [28], although not guaranteed. NVivo V.12 Pro software will be used to support coding consistency, and charting the anonymised participants' data excerpts in Microsoft Excel will aid verifying the plausibility of themes identified.

Objective 2 output: a summary report of the mapping and qualitative findings to support codevelopment of intervention goals and programme theory

Objective 3. Codevelopment of intervention goals and programme theory

Data synthesis. Data will be synthesised in narrative summaries focusing on intervention needs of adolescents, and the strengths and limitations of behaviour change approaches relevant to stated needs. To aid understanding for non-academics, visual summaries (including videos, which the team has experience in making accessible to those in low resource setting) will be produced by the public involvement group, supported by the researchers and other collaborators. The data sources include: (1) data from objective 2 (mapping, focus group and interview analysis); (2) existing literature, including our existing compendium of effective evidence-based behaviour techniques, community engagement and health promotion guidelines [19]; and (3) available intervention components and other resources that can potentially be adopted/ adapted (with permission), in addition to developing our own novel components and resources.

Theory mapping**.** The narrative summaries will be shared with the consortium for review and feedback and final adjustments will be made in the bi-monthly/ public involvement group meetings, months 5-6. Findings will be mapped to and develop the current theoretical framework.

Objective 3 output: A systems map and logic model of change, system levers and influencing factors

2.2 Stage 2: Co-designing and creating:

Objective 4. Generate ideas about intervention components and features

The core team will generate ideas about intervention components and features using the logic model to produce a theorised list of the components, their content and delivery, consistent with the underpinning theory.

Objective 4 output: A theorised list of potential components, content, and mode/s of delivery.

Objective 5: Make decisions on the intervention content, format, and delivery

We will achieve consensus on intervention content, format and delivery, process and outcome evaluation items and methods for the future research phases at the second coproduction workshop and ancillary meetings for specialist input (e.g. from the health economists).

A one day community dissemination event will also be organised in one of the participating villages, with approximately 100 participants including potential intervention beneficiaries, other community members, the consortium team, and additional relevant stakeholders from the wider system. Interim findings will be shared, including via '*Keneleng'* (informal women's singing and drama groups). Attendees will be encouraged to share their views on the plans for content, format and delivery of the intervention which will be incorporated into the documentation stage.

Objective 5 output: Intervention content, format, and delivery decisions; prototype materials; a formal implementation plan

2.3 Stage 3: Documentation, evaluation planning and dissemination:

Objective 6: We will document and disseminate an intervention manual and other outputs suitable for diverse audiences, including an infographic for adolescents and communities, in addition to publications and presentations. These are the key benefits from this project. The transparent research process, careful documentation and varied dissemination of outputs will maximise the impact of the project outcomes which are to: i. Add new knowledge to the evidence base for what is likely to be effective in addressing malnutrition among adolescents in The Gambia; and ii. Use the outputs to refine and test the intervention (see future development phase).

Objective 6 output: An intervention manual and a co-designed evaluation plan

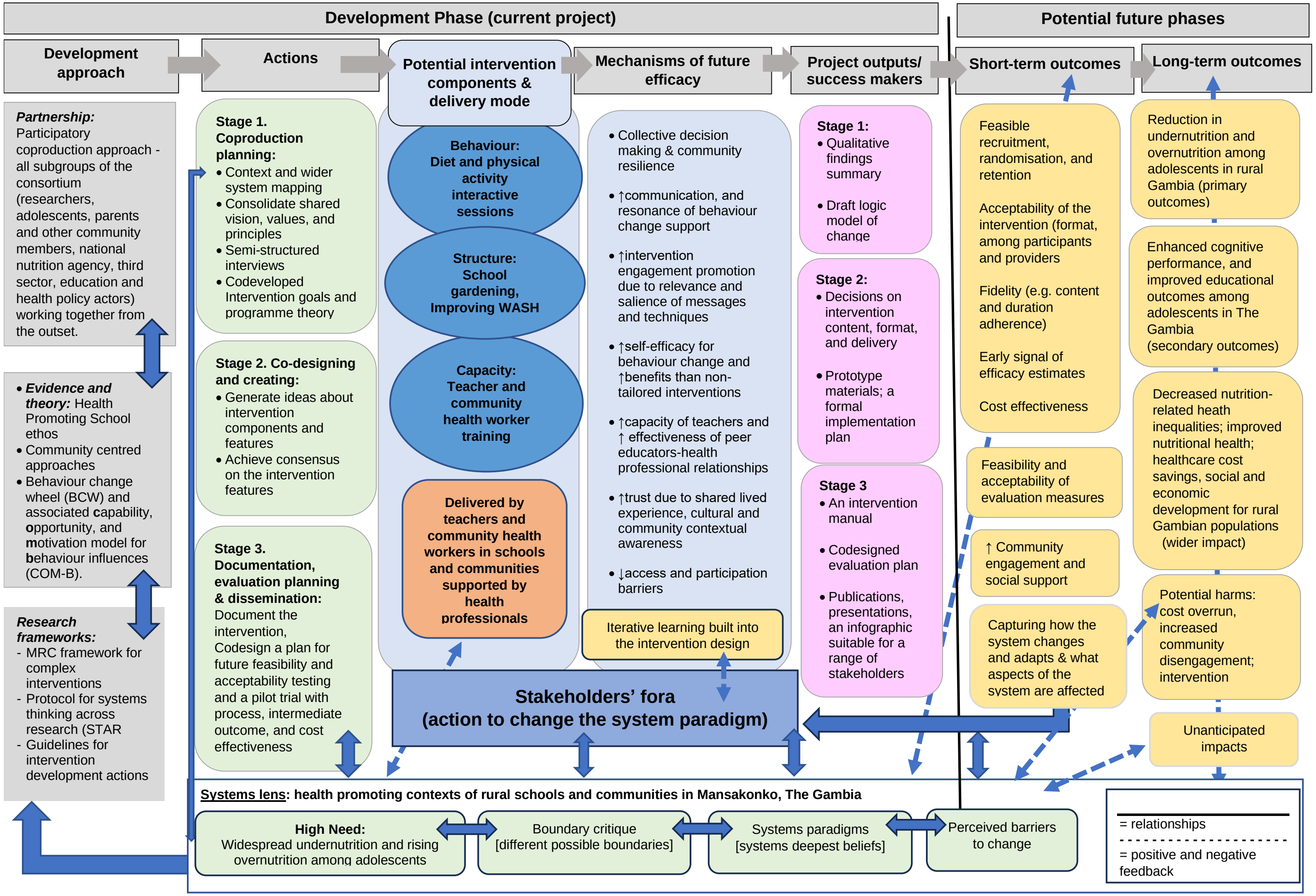

Figure 1: Preliminary systems map and logic model

Month:		Preliminary preparation		S	O	N	D	J	F	M	A	M	J	J	A
Task	Staff			2024				2025							
**PROJECT MANAGEMENT (months 0-13):**															
Weekly meetings of the core research team (project lead [PL], researcher co-lead (RcL); online/ in person/ hybrid)	MM, HJB, (A-MB 1x per month)			x4	x4	x4	x4	x4	x4	x4	x4	x4	x4	x1	x4
Set up the data and administrative systems, prepare materials, and draft the ethics application	MM, HJB														
Public involvement in research (PIR) group meetings to establish and provide support for their project partnership (in person)	PIR group, HJB/FK														
Data collection training for two fieldworkers (FWs)	FWs[1], MM, HJB														
Consortium hybrid (in-person and online) meetings (approx. bi-monthly) to ensure coproduction	All[2]														
Coproduction consortium workshop 1 (in-person)	MM, HJB, PIR group, Gambia partner, and collaborators														
**STAGE 1 Coproduction planning (months 0-6):**															
Obtain consortium input on ethics/ data collection materials; submit application	All														
Conduct context and wider system mapping	HJB, FWs, MM														
Recruit qualitative focus group and interview participants	HJB, FWs														
Conduct qualitative focus groups and interviews	HJB, FWs														
Analyse interviews and produce interim and *summary reports*[3] for consortium input	MM, HJB														
Conduct and share data synthesis (qualitative data; literature; existing resources)	MM, HJB														
Jointly define target behaviours using electronic feedback on the data synthesis summaries and bi-monthly consortium/ PIR meetings	MM, HJB, input from All														
Use the input received to map ideas to a *draft logic model of change*	MM, HJB														
**STAGE 2: Co-designing and creating (months 7 to 9):**															
Use electronic feedback, bi-monthly consortium/ PIR meetings to coproduce a theorised list of *intervention components, content, and delivery*	MM, HJB, input from All														
Organise and hold the bi-monthly consortium/ PIR meetings to consolidate views	All														
Develop the *prototype intervention materials*	MM, HJB														
Achieving consensus on intervention features through co-production workshop 2 and the community event	All														
Devise a *formal implementation plan*	MM, HJB; input from All														
**STAGE 3: Documentation, evaluation planning & dissemination (months 10 to 13):**															
Document a description of the intervention and a *formal feasibility and acceptability study and pilot trial plan*	MM, HJB, input from All														

Figure 2: Gantt chart

3. Future development phase

3.1 Type of intervention, form and function

The intervention is likely to include nutrition-specific components (e.g. food supplementation interactive nutrition education; tailored physical activity [PA] sessions). Inclusion of nutrition-sensitive components resulting in changes to wider school and community contexts (e.g. school gardening; improving WASH facilities; teacher and community health worker training) is also indicated by the existing evidence, public contribution and theoretical approach. Facilitating acceptable access to wider programmes (such as anti-parasitic treatment) is necessary, as lack of access is a significant barrier to and diminishes the impact of nutrition-specific population-level activities [20]. Including actions that change the system's goals (i.e. the system's underlying paradigm), such as maintaining stakeholder fora as part of the intervention, is consistent with our systems lens [22]. The intervention will be delivered, through partnership, by school personnel, community health workers, and health professionals.

3.2 Programme theory

Formulating the programme theory of change will be informed by the STAR protocol [22]. This will involve the development and refinement of the mid-range theory and frameworks (HPS; community-centred approach, behaviour change theory) to incorporate feedback loops to suggest how context and intervention interact to produce outcomes. Anticipated outcomes are primarily decreased under- and overnutrition for rural Gambian adolescents, and changes in systems goals; improved cognitive/educational performance are secondary outcomes. Refinement of the preliminary systems map and logic model (Figure 1) is one of the objectives and outputs of Stage 1 of the current project.

3.3 Intervention development and pathway to the next stage

Progression to the next phase will be indicated by success in producing outputs from this current development phase (co-produced systems map and logic model of change; an intervention manual describing the intervention structure, components, and delivery; and exemplar resources). Assuming success, the next phase will be observational feasibility and acceptability testing (intervention refinement), which may then be linked to a pilot cluster-randomised trial. Mixed methods process evaluation, intermediate outcomes, and cost-effectiveness evaluation will aid insight into success or failure and/ or unexpected consequences. Diverse evaluation methods will be used to ensure that, as well as quantifiable elements, systems concepts: 'emergence' [emergent properties that are features of the system as a whole]; 'feedback'; and 'adaptation' [change of system behaviour in response to an intervention] [9] are incorporated. Evidence of feasibility and effectiveness will lead to further refinement, optimisation of intervention efficiency, an implementation, upscaling, and sustainability strategy. We will review emergent *wholistic* approaches integrating implementation and systems frameworks [29;p12] in planning future research phases.

4. Team capability

Team composition

Core research team, Leeds Beckett University

1. Prof Maria Maynard (MM), Project Lead. Professor of Health Inequalities.
2. Mrs Haddy Jallow Badjan (HJB), Researcher Co-lead. Post-doctoral Researcher, Global Health.
3. Prof Anne-Marie Bagnall (A-MB), Project Co-lead. Professor of Health & Wellbeing Evidence.

Public involvement

Approx. 10-12 members of existing public contributors in the participating rural areas of the Gambia to include 4-6 adolescents, two parents and teachers, two community leaders; and will be co-ordinated by Ms Fatoumatta Kassama (FK), Founder and CEO of Girls Pride, The Gambia.

Project partner

Mrs Haddy Crookes (HC), Project partner contact. Senior Programme Officer, National Nutrition Agency (NaNA), The Gambia.

Academic collaborators - The Gambia

1. Dr Ramatoulie Janha (RJ), MRC Unit The Gambia at LSHTM.
2. Dr Hassan Njie (HN), Health economist, Part- Time Lecturer, Department of Public and Environmental Services, The University of The Gambia, and Director of Services, National Health Insurance Authority, The Gambia.
3. Dr Thomas Senghore (TS). Senior Lecturer, Public Health (epidemiology), University of The Gambia.
4. Dr Haddy Tunkara Bah (HTB), Senior Lecturer Nursing and Reproductive Health, University of The Gambia.

Academic collaborators – UK

1. Dr Tanefa Apekey (TA). Senior Lecturer Public Health Nutrition, University of Sheffield.
2. Dr Oritseweyinmi Orighoye (OO). Associate Lecturer Health and Care Management, University of Arden.
3. Mrs Tayamika Zabula (TZ). Research Fellow Economic evaluation, health economics & econometrics, University of Leeds.

Non-academic collaborators

1. Mr Abdou K. Jallow (AJ), Head of Adolescent Unit, Reproductive, Maternal, Neonatal, Child and Adolescent Health (RMNCAH) Unit, Ministry of Health, The Gambia.
2. Mr Momodou Jallow (MJ), Regional Education Director, Regional Education Directorate Six, Mansakonko LGA, The Gambia.

Contributions to the generation of new ideas, tools, methodologies or knowledge

The team's outputs are generated from £8.7M of research and capacity building grant capture from research councils and other major funding bodies (e.g. MRC, National Institutes of Health (US); National Institute of Health Research, Public Health England (UK)), including as project leads (MM, A-MB, TA, TS, HTB). Of primary significance is how we have advanced integrating management of acute malnutrition into community services (HC); experience-based evidence of the need for culturally tailored nutrition programmes (e.g. Re:mission, MM, TA, TZ); centred the voices of least heard ethnic groups in coproduced health programmes (Health Connections, MM, TA, HJB), and developed methodologies and frameworks for robust whole systems action to improve population health (A-MB). Novel observational research among adolescents (HJB, HTB, TS, HC, FK) provides much of the intelligence informing the need for the development, implementation and evaluation of interventions in The Gambia. Team members also combine skills and expertise in economic evaluation of interventions (TZ) with broader health economic topics such as universal health coverage (HN) and impact of agricultural subsidies on nutrition (TZ), informing on the role of wider economic determinants in the sustainability of health interventions. AJ (National RMNCAH policy), MJ (Education for All National Action Plan), and HC (scaling up nutrition (SUN) Network) directly shape health and education policy in The Gambia, translating research into real world benefit. Through the applied emphasis of our research, we actively conduct work of policy impact (e.g. informing policy actions for addressing obesity among high risk children, and local authority food strategy TA, MM; REF 2021 Centre for Health Promotion Research [CHPR] impact case study, A-MB). Collectively we are skilled in diverse theoretical frameworks, epidemiological and social science approaches, and thus the quantitative, qualitative, and mixed methods crucial to systems-informed intervention development. Creative educational tools developed within the team include photography (RJ), videos (TA, FK), and gaming (FK), which will be applied in the current project. Policy briefs, reports, and presenting to a range of audiences complement the team's 315 peer-reviewed publications.

The development of others and maintenance of effective working relationships

Strategic leadership is evident in the core team's roles within research groups (Migrant Health Research group, MM) and centres of excellence (CHPR, A-MB). Existing international collaborations and links throughout the wider team are directly relevant to adolescent nutritional health (e.g. the GCRF funded Ghana-Nigeria-UK network, co-led by MM, and including other team members TA, OO; and membership of the TALENT consortium, RJ). This furthers the opportunity for building on existing formal and informal evidence bases, and linking the current work with complementary work of other teams.

Supervision of 22 master's students by HTB and TS has contributed to research capacity building among public health practitioners in The Gambia, with four former students progressing to senior roles after their studies. MM, A-MB, and TA have developed research skills and supported progression of researchers to independent status through supervising seven PhD students to completion (six retained in health research careers; three from LMICS, building research capacity in their home countries); and supported consolidation of national and international research reputations through line-management or mentoring of four post-doctoral fellows and six early- to mid- career researchers (including women in to STEM careers mentoring).

Contributions to the wider research and innovation community

We demonstrate our commitment to research excellence through reviewing grant proposals (e.g. for MRC, NIHR, ESRC: MM, TA, A-MB) and journal editorial roles (e.g. Frontiers in Public Health, MM). We also demonstrate our collegiality with a range of committee roles in our organisations; and voluntary roles including charity trusteeships (MM, A-MB, FK, OO, TA).

Our wider contributions include commitment to antiracist research and practice, and decoloniality in global health research, evident in our research priorities.

Contributions to broader research/innovation-users and audiences and towards wider societal benefit

Coproduction is embedded throughout the team's research and practice priorities, exemplified by public and policy engagement activities in The Gambia, Ghana, and Nigeria. With our UK experience, this provides LMIC-UK reciprocal learning on inclusive public contribution and engagement. FK's grassroots, award winning innovation has significantly reduced lost education and PA opportunities among girls in The Gambia through transformative education of over >20,000 children and adults on menstrual health and hygiene management and distribution of >15,000 eco-friendly and reusable sanitary products. Previous development and use of creative methods of engagement and dissemination (drama, video/photography, gaming and singing (FK, RJ, OO), are incorporated into the current development activities.

5. Data management and sharing

1.1 Type of study

Mixed methods intervention development study.

1.2 Types of data

Observations: photography and completed checklists

Quantitative questionnaire data: short questionnaires from key informants; socio-demographic questionnaires from adolescent participants.

Qualitative data: focus groups and interviews among the adolescent participants.

1.3 Format and scale of the data

All data will be stored in a format that enables sharing and long-term validity of data, and are unlikely to exceed 1GB.

Survey data will be stored as a .csv file and in SPSS statistical software package files

Interviews – anonymised transcripts will be stored as .docx files.

Anonymised transcripts will also be uploaded and stored as an QSR NVIVO file (.nvp)

Creative methods will be stored as picture files.

Checklists and fieldnotes will be stored as pdf files.

Data collection / generation

2.1 Sources of data

Research methods are researcher administered questionnaires, qualitative data collection via interviews and focus groups by and experienced interviewer, reviews of existing literature and resources. Rigorous, documented methodologies used throughout.

2.2 Data quality and standards

Consistency and quality of data collection will be ensured through standardised data collection (questionnaire, interviews schedule), standardised recording (interviews – voice recorders, verbatim transcription), and data entry validation/cleaning (survey).

2.3 Consent for data sharing and re-use

The data will be suitable for sharing. It will be anonymised, so will not provide a risk of breaching confidentiality. The data will have utility for other researchers interested in public health intervention development. Participants will be informed about how their data will be used, stored, shared, disposed of, including who will have access to their data. Participant agreement with their use of data will be certified on the signed consent and assent forms. Additionally, information will be provided verbally.

Data management, documentation and curation

3.1 Managing, storing and curating data

Data will be stored on Leeds Beckett University's (LBU; lead institution) OneDrive system, managed by the LBU IT Service, which meets UK and EU data security regulations (including GDPR). The data are stored simultaneously on six data centres to allow for recovery, and the files will be encrypted at rest and when in transit to the cloud. The files are maintained by the PL. As this is a centrally managed storage repository, this ensures no data is lost. A back-up of the data is downloaded on a monthly basis to avoid data loss and stored on a password-protected area of the university H drive.

3.2 Metadata standards and data documentation

Descriptions of the methods used to generate the data, descriptions of variables and analytical and procedural information.

3.3 Data preservation strategy and standards

Accumulated (anonymised/summarised) research data will be deposited in LBU's Data Repository (https://figshare.leedsbeckett.ac.uk) where it will be open access and protected under a CC BY License. Any data deemed identifiable data will be kept for no longer than is necessary to meet the purposes for which the data will be processed.

Data security and confidentiality

4.1 Formal information/data security standards

LBU does not have ISO 27001. As all the research data will be anonymised the risks are low. All data will be stored securely on LBU servers. LBU will implement a Collaboration agreement with all relevant parties which will detail the technical security requirements.

4.2 Main risks to data security and how they will be managed

Contact information provided for organising interviews and focus groups will be stored securely, separately from research data only accessible to the PI and RcL to assign unique codes to anonymise the data, link data (such as questionnaire and interview data) or remove data at participants request (up to the time limits stated on the PIS). Similarly only the PI and RcL will have access to information such as ethnicity, and only available to others in summary form. Data will be stored according to data security regulations (including GDPR), as noted above. Data security risks are low as the research data will be anonymised. Main risks to data security: data getting lost and/or damaged and this is mitigated against by the data storage

procedures outlined. Access to data by the wider team (including overseas) will be of anonymised/ summarised data only, abiding by the data management policy.

Data sharing and access

Anonymised research data will be deposited in LBU's Data Repository (https://figshare.leedsbeckett.ac.uk) where it will be open access and protected under a CC BY License.

5.1 Suitability for sharing

The data will be suitable for sharing with the team and external users as it will be anonymised and or summarised, and so will not provide a risk of breaching confidentiality. Re-identification will not be possible by these users as they will not have access to the information required for this as stated in 4.2.

5.2 Discovery by potential users of the research data

Accumulated (anonymised) research data will be deposited in LBU's Data Repository (https://figshare.leedsbeckett.ac.uk/) where it will be open access and protected under a CC BY License. Further, all research outputs will be open access in the LBU Library Repository (https://eprints.leedsbeckett.ac.uk/). If academic journal articles are embargoed, an "accepted for publication" version will be obtainable from the repository until the published version is available.

5.3 Governance of access

Anonymised research data will be deposited in LBU's Data Repository (https://figshare.leedsbeckett.ac.uk) where it will be open access and protected under a CC BY License.

5.4 The study team's exclusive use of the data

The research team will have exclusive access to the data for a maximum of 12 months following the completion of the project, to allow the team to produce all proposed outputs. After this, the data will be deposited into the open-access data repository.

5.5 Restrictions or delays to sharing, with planned actions to limit such restrictions

As part of the consent process, data sharing plans will be clearly set out and current and potential future risks associated with this explained to research participants.

5.6 Regulation of responsibilities of users

Data will be managed in line with LBU data management policy, held securely and only accessible to members of the research team, until it is made Open Access. Data sharing agreements will be put in place between consortium members for the duration of the project.

5.7 Working with overseas collaborators or data users

Sensitive information data will not be shared with overseas collaborators and partners. Access will only be granted to anonymised/ summarised information, and according to the data management policy.

Responsibilities

LBU IT Security team for data security

LBU Research Information and Governance for study-wide data management and quality assurance of data.

https://www.leedsbeckett.ac.uk/our-university/public-information/university-policies/

Relevant institutional, departmental or study policies on data sharing and data security

https://www.leedsbeckett.ac.uk/our-university/public-information/university-policies/

Author of this data management plan

Haddy Jallow Badjan (RcL), h.jallow-badjan@leedsbeckett.ac.uk and Maria Maynard (PL).

6. Ethics and responsible research and innovation (RRI)

We will adhere to the MRC guidelines for ethics and approval, and the regulations of the required approving bodies (see Research involving human participation).

Participation in the research will be voluntary and to ensure informed consent respondents will receive a participant information sheet (PIS) about the study, prior to deciding to take part. Consent will be certified with a signed consent form (parents; other adults) and assent form (children). Information will be provided in varied formats: written, other visual (e.g. diagrams, spoken, and spoken in Mandinka, the predominant language) to accommodate diverse literacy needs. The PIR group coordinator (Kassama) has links with The Gambia Organization of the Visually Impaired, who will provide braille versions of written materials without charge if required.

Participants will have the right to not answer questions they are not comfortable with, and to withdraw from the data collection process (observations, focus groups, interviews, questionnaire completion) without giving a reason. Data can be withdrawn after contributing to data collection up to approximately February 2025 when analysis and synthesis of the data will have commenced. Focus groups and interviews will be recorded with permission, and as certified on the consent form.

Risk to participants is not anticipated to exceed that of day to day living. It is possible that thinking or talking about the topics within the discussions about behaviour or attitudes regarding eating, activity, and other health related behaviour (e.g. accessing healthcare) including associated stigma and cultural norms may generate difficult feelings and/or the need to discuss them further. The research team has the expertise to conduct engagement with participants and data collection with sensitivity, and activities will be stopped if there are signs of distress. Participants will also be signposted on the PIS and verbally to additional sources of culturally relevant information and support, and will be advised to speak with others e.g. community health worker if they have any concerns which cannot be addressed by general information. The research team have considerable experience in working with a range of participants (including young people, and those that may be potentially vulnerable). There is no direct benefit to individuals in taking part; however, the involvement of volunteers is essential for the study to take place. Therefore all participants will contribute to the outputs from the study and to the potential long-term benefits of those outputs. All participants will receive a thank-you gift at a level and nature that is not likely to coerce subjects into taking

part, but will demonstrate the research team’s acknowledgement of participants’ time and contribution of their views.

Risk to researchers is also minimal, but as data collection will be carried out in the community, these sessions will be conducted by a team of three researchers, thereby avoiding any lone researcher working, and ensure safeguarding of participants.

All data will be stored ethically and securely as described in the data management plan (DMP). The DMP will also be a component part of the applications for ethical approval.

7. Research involving human participation

Ethical approval will be obtained from the Research Ethics Committee, School of Health, Leeds Beckett University, and The Gambia Government/MRC Unit The Gambia at LSHTM Joint Ethics Committee. Permission will also be sought from the Regional Education Directorate and participating village Chiefs in Mansakonko LGA. We have prior experience of all of these procedures.

Objectives 2i, 2ii, and 5 will involve the inclusion of human subjects contributing data to the project.

Objective 2i: Mapping context.

Understanding context is essential to the theoretical underpinning and proposed intervention development actions. Data collection will include observation sessions and brief questionnaires completed by key informants. The process for selecting the schools and communities to be mapped, as described in the project Approach ensures that the diverse contexts, people and relationships relevant to the study objectives are captured. The purposive, convenience sample of approximately n=50 key informants from the school and community settings, will permit stakeholder subgroup analysis (teachers, parents, community health workers, other community members, and system stakeholders identified through snowball sampling). Participants from a range of roles will be included, and we will aim to actively recruit those who are typically excluded from power and decision making.

Objective 2ii: Understanding experiences, perspectives, and needs of the target population

Understanding and centring the life experiences and views of the target populations is an essential action in new intervention development. We will conduct semi-structured focus group discussions and interviews to obtain views relevant to project objectives. Focus group participants will be adolescents in the participating schools, and interviews with those not attending school. Although poverty is common throughout the setting, there are subtle socio-demographic (e.g. family size, parental occupation), as well as ethnic and religious differences between those attending the two main types of school, and between those attending and not attending school. Ways of engaging with school education also varies between school types

(e.g. later age of starting school, staying in school after school leaving age, attending for part of the day in madrassas). We will also actively involve adolescents marginalised through not attending school. The target sample of 48-70 adolescents from the planned focus groups and interviews will provide breadth of understanding of the phenomenon of interest while still permitting in-depth inductive analysis. In turn, this aids the transferability of the findings and interpretations to other contexts or settings beyond this research project [28] [36]. Theoretical qualitative researchers suggest that reaching saturation is also likely to occur with the planned sample size [29] [37]. Saturation will be determined by iterative analysis alongside data collection, and using a framework method to chart the anonymised participants and themes generated.

We will draw on the team's community links to liaise with community gateway persons (such as representatives from the VDC), prioritising oral communication and face to face meetings and to support raising awareness of the study in schools and communities. This will be part of the approval process, and is one that is sanctioned by adolescents in previous research [5,21] [3, 9]. Community gateway persons will therefore be important links with participants; however, recruitment of the individuals will be down to individual's (and their parents') choice. Mechanisms for registering interest (e.g. signup sheets, contact numbers), and informed consent procedures will be in place. Recruitment will continue iteratively until proposed numbers in categories have been reached.

Objective 5. Make decisions on the intervention content, format and delivery

In order to refine the decisions on the features of the intervention being developed and prior to documenting the intervention that is being developed we will gain potential intervention beneficiaries (adolescents') views at a community event. The event will be held in one of the villages in the research setting. The project research participants will be invited as well as other adolescents, families and other community members, the consortium team, and additional relevant stakeholders from the wider system. Observation, photography, and brief questionnaires will be used to obtain participants views on the intervention plan.

Event participants will therefore constitute a convenience sample. The sample size cannot fully be predetermined; however, from our experience with research in these communities, we anticipate 80-100 attendees and an estimated 30% will complete questionnaires, yielding data for a sample of approximately 30 adolescents, women and men to add to the observations.

8. References

1. Fall, C.H., et al. Anthropometric nutritional status, and social and dietary characteristics of African and Indian adolescents taking part in the TALENT (Transforming Adolescent Lives through Nutrition) qualitative study. Public Health Nutrition, 2021. 24(16): p. 5249-5260.(1)
2. Jallow-Badjan, H., et al. Prevalence and Factors Associated with Thinness and Overweight/Obesity Among Secondary School Adolescents. A Cross-sectional Study. Public Health, 2020. 6(3): p. 164-172. (2)
3. Tunkara-Bah, H., H.J. Badjan, and T. Senghore. Dietary factors associated with being overweight and obese among school-going adolescents in Region One, The Gambia. Heliyon, 2021. 7 (3).
4. Janha, R.E., et al. Exploring influences on adolescent diet and physical activity in rural Gambia, West Africa: food insecurity, culture and the natural environment. Public Health Nutrition, 2021. 24(16): p. 5277-5287. (4)

5. Jallow Badjan, H., T. Apekey, and M. Maynard. Health and nutrition survey among girls in The Gambia: A mixed-methods pilot study. Under review. (5)
6. Gambia Bureau of Statistics (GBoS) and ICF. The Gambia Demographic and Health Survey 2019-20. 2021, Banjul, The Gambia and Rockville, Maryland, USA: GBoS and ICF. (6)
7. Hennig, B.J., et al. Cohort profile: the Kiang West Longitudinal Population Study (KWLPS)—a platform for integrated research and health care provision in rural Gambia. International Journal of Epidemiology, 2017. 46(2): p. e13. (7)
8. Moore, S.E. Using longitudinal data to understand nutrition and health interactions in rural Gambia. Annals of Human Biology, 2020. 47(2): p. 125-131. (8)
9. Skivington, K., et al. A new framework for developing and evaluating complex interventions: update of Medical Research Council guidance. BMJ, 2021. 374: p. n2061. (9)
10. Skivington, K., et al. Framework for the development and evaluation of complex interventions: gap analysis, workshop and consultation-informed update. Health Technology Assessment, 2021. 25(57): p. 1. (10)
11. O'Cathain, A., et al. Taxonomy of approaches to developing interventions to improve health: a systematic methods overview. Pilot and Feasibility Studies, 2019. 5(1): p. 1-27. (11).
12. Rod, M.H., et al. Promoting the health of vulnerable populations: Three steps towards a systems-based re-orientation of public health intervention research. Health & Place, 2023. 80: p. 102984. (12)
13. Langford, R., et al. The health promoting schools framework: known unknowns and an agenda for future research. Health Education & Behavior, 2017. 44(3): p. 463-475. (13)
14. WHO. Health Promoting Schools. Available at: https://www.who.int/health-topics/health-promoting-schools#tab=tab_1. [Accessed 01/03/24]. nd. (14)
15. South, J., et al. An evidence-based framework on community-centred approaches for health. Health Promotion International, 2019. 34(2): p. 356-366. (15)
16. Public Health England. Community-centred public health: Taking a whole system approach. Available from: https://assets.publishing.service.gov.uk/media/5e184c78e5274a06b1c3c5f9/WSA_Briefing.pdf [Accessed 21/2/24]. 2020. (16)
17. Sniehotta, F.F., et al. Complex systems and individual-level approaches to population health: a false dichotomy? The Lancet Public Health, 2017. 2(9): p. e396-e397. (17)
18. Michie, S., M.M. Van Stralen, and R. West. The behaviour change wheel: a new method for characterising and designing behaviour change interventions. Implementation Science, 2011. 6(1): p. 1-12. (18)
19. Maynard, M.J., et al. 'Health Connections': study protocol for the development of a coproduced, community-based diet, physical activity, and healthy weight intervention for UK Black and Asian adults. BMJ Open, 2023. 13(9): p. e073750. (19)
20. Jallow Badjan, H. Social determinants of the nutritional status of female adolescents in The Gambia. PhD thesis. School of Health. 2023, Leeds Beckett University: Leeds. (20)
21. Bagnall, A.-M., et al. Whole systems approaches to obesity and other complex public health challenges: a systematic review. BMC Public Health, 2019. 19: p. 1-14. (21)
22. Knai, C., et al. Learning from the CO-CREATE project: A protocol for systems thinking across research (STAR). Obesity Reviews, 2023. 24: p. e13624. (22)
23. O'Cathain, A., et al. Guidance on how to develop complex interventions to improve health and healthcare. BMJ Open, 2019. 9(8): p. e029954. (11) (23)
24. Bröer C, et al. Recruiting and engaging adolescents in creating overweight and obesity prevention policies: the CO-CREATE project. Obesity Reviews. 2023 Feb;24:e13546.

25. Klepp, K.I., et al. Overweight and obesity prevention for and with adolescents: the "confronting obesity: co-creating policy with youth"(CO-CREATE) project. Obesity Reviews, 2023. 24: p. e13540. (25)
26. Adler, K., S. Salanterä, and M. Zumstein-Shaha. Focus group interviews in child, youth, and parent research: An integrative literature review. International Journal of Qualitative Methods, 2019. 18: p. 1609406919887274. (26)
27. Braun, V. and V. Clarke. Thematic analysis: a practical guide 2022, London: SAGE Publications. (27)
28. Guest, G., A. Bunce, and L. Johnson. How many interviews are enough? An experiment with data saturation and variability. Field methods, 2006. 18(1): p. 59-82. (28)
29. Whelan, J., et al. Combining systems thinking approaches and implementation science constructs within community-based prevention: a systematic review. Health Research Policy and Systems, 2023. 21(1): p. 85. (29).

9. Example Appendices

[N.B. example appendices are presented in line with the guidance. A full set of appendices have been uploaded within the online application]

1. Observation checklist

Health-Secure Partnership Project

Context mapping

OBSERVATION CHECKLIST

**Date:**
**Time:**
**Village code:**
**Community code:**
**School present:** Yes ☐ No ☐
**School code:**
**Length of time spent in observation:……..hrs…….mins**

Part 1: School food environment

1.1. Describe any food outlets in or near the school grounds

Food outlet	Type	Location	Popular purchases
1.			
2.			
3.			
4.			
5.			

1.2. Describe the places in or near school where pupils eat and/or drink

1.3. Describe the food related activities in or near school (e.g. sharing/ not sharing food; types of food and drinks consumed, etc.)

Part 2: School physical activity (PA) environment

2.1. Describe the places where pupils do PA in or near school

2.2. Describe the PA related activities in or near school (e.g. playing in groups/ alone; sport; ball games; skipping; running; walking etc.)

PART 3: WASH IN SCHOOLS CHECKLIST (core questions)

**GENERAL INFORMATION**
SCHOOL NAME: ____________________________________ DATE: ________________<br>TYPE OF SCHOOL: ☐ Pre-school (Ages 4-5) ☐ Primary School (Ages 6-12) ☐ Secondary School (Ages 13-19) ENROLMENT: Total __________ Boys______________ Girls___<br>STAFF: Total_________ Full-time_____________ Part-time______________
**CORE JMP QUESTIONS - WASH IN SCHOOLS**
W1. What is the main source of drinking water provided by the school? (check one - most frequently used)

	**Improved**<br>☐ Piped<br>☐ Tube well/Borehole<br>☐ Protected dug well<br>☐ Protected spring<br>☐ Rain water<br>☐ Tanker truck<br>☐ Other:________<br>**Unimproved**<br>☐ Unprotected dug well<br>☐ Unprotected spring<br>☐ Surface water (River/Lake/Canal)<br>☐ No water source
W2.	Is drinking water from the main source currently available at the school?<br>☐ Yes ☐ No
S1.	Type of toilets/latrines (select one – most common):<br>**Improved**<br>☐ Flush/Pour-flush to sewer<br>☐ Flush/Pour-flush to tank or pit<br>☐ Flush/Pour-flush to open drain<br>☐ Pit latrine with slab/covered<br>**Unimproved**<br>☐ Pit latrine without slab/open<br>☐ Bucket<br>☐ Hanging toilet/latrine<br>☐ None
S2 & S3 (alt)	How many toilets/latrines are at the school (insert number)?
H1.	Are there handwashing facilities at the school?<br>☐ Yes ☐ No
H2.	Are both soap and water currently available at the handwashing facilities?<br>☐ Yes, water and soap<br>☐ Water only<br>☐ Soap only<br>☐ Neither water or soap

WASH IN SCHOOLS CHECKLIST – NOTES

CORE JMP – WASH IN SCHOOLS QUESTIONS	
W1	☐*If there is more than one source, the one used most frequently for drinking water should be selected. If children need to bring water from home because water is not provided by the school, "no water source" should be selected. Response options should be modified to reflect the local context and terminology such that respondents are able to clearly understand each one, and they are able to be categorized as improved, unimproved or no water source.*
W2	☐*To be considered available, water should be available at the school at the time of the survey or questionnaire, either from the main source directly or stored water originally from the main source.*
S1	☐*If more than one type is used, the most common type of student toilet/latrine should be selected. Response options should be modified to reflect the local context and terminology such that responses are able to be categorized by improved, unimproved or none.*

S2 & S3 alt	☐*Only count toilets/latrines that are usable at the time of the survey or questionnaire, where "usable" refers to toilets/latrines which are*<br>*(1) available to students (doors are unlocked or a key is available at all times),*<br>*(2) functional (the toilet is not broken, the toilet hole is not blocked, and water is available for flush/pour-flush toilets), and*<br>*(3) private (there are closable doors that lock from the inside and no large gaps in the structure) at the time of the questionnaire or survey. If any of these three criteria are not met, the toilet/latrine should not be counted as usable. However, lockable toilets may not be applicable in pre-primary schools.*<br>☐*Single-sex toilets means that separate girls' and boys' toilets are available at the school, or it is a single-sex school and has toilets.To be considered separate, facilities should provide privacy from students of the opposite sex, but this definition should be further defined based on local context, as needed. For schools that have separate shifts for girls and boys (i.e. girls attend the school at a separate time from boys), depending on local culture, the response could be "yes" since at the time of use, the toilets are only for girls. This question may not be applicable in pre-primary schools.*<br>☐*It should meet the following conditions:*<br>*(1) can be accessed without stairs or steps,*<br>*(2) handrails for support are attached either to the floor or sidewalls,*<br>*(3) the door is at least 80 cm wide, and*<br>*(4) the door handle and seat are within reach of people using wheelchairs or crutches/sticks.*
H1	☐*A handwashing facility is any device or infrastructure that enables students to wash their hands effectively using running water, such as a sink with tap, water tank with tap, bucket with tap, tippy tap, or other similar device. Note: a shared bucket used for dipping hands is not considered an effective handwashing facility.*
H2	☐*To be considered available, water and soap must be available at one or more of the handwashing facilities at the time of the survey or questionnaire. If girls and boys have separate facilities, soap and water should be at both. Soapy water (a prepared solution of detergent suspended in water) can be considered as an alternative for soap, but not for water, as non-soapy water is needed for rinsing. Surveys may choose to add other response categories for ash or alcohol hand rub, but these should be kept as separate categories from soap to support SDG monitoring.*

Part 4: Village community food environment

4.1. Describe any food outlets in the village community

Food outlet	Type	Location	Popular purchases
1.			
2.			
3.			
4.			
5.			
6.			
7.			
8.			

4.2. Describe the places where adolescents and/ or other village members eat and/or drink

4.3. Describe the food related activities (e.g. sharing/ not sharing food; types of food and drinks consumed, etc.)

Part 5: Village community (PA) environment

5.1. Describe the places where adolescents and/or other community members do PA

5.2. Describe the PA related activities (e.g. playing in groups/ alone; sport; ball games; skipping; running; walking etc.)

5.3 Provide an overall summary description of the general village environment including places for social gatherings (e.g. community centre/s; places of worship)

PART 6: Health care facilities

6.1. Describe any healthcare facilities in the village community

Health care facilities	Type	Location	Accessibility
1.			
2.			
3.			
4.			
5.			
6.			
7.			
8.			

6.1. If there are no healthcare facilities in the village community, note the distance to the nearest facility from the village

………..KMs

PART 6. Sketch a basic map of the village, noting key elements: main roads, school/s, food outlets, areas for outside play, other gathering places (e.g. community centres, places of worship)

PART 7: Any other observations.

7.1 Note any final observations not covered in the previous sections

2. Adolescent questionnaire

Office use only
ID NO:

This questionnaire is part of the **Health-Secure Partnership** project**.** Kindly read each question and the answer options carefully before answering. You can ask for clarification regarding any question at any time. We can also explain the questions in English or Mandinka.

We would like you to complete **all of the questions** unless otherwise stated. However, you are free to omit any question you do not want to answer.

Please tick ✓ **ONE** box for each question (unless otherwise instructed), or write in your answer where appropriate (look for this symbol ✐).

Section 1: About you

1. How old are you? Age (in years): ✐

2. What is your country of birth? Country name: ✐

3. Are you a boy or a girl?

1. Boy [ ]	2. Girl [ ]

4. What is your religion?

1. Muslim [ ]	2. Christian [ ]	3. Other, please specify: ✐

5. What is your tribe?

1. Mandinka [ ]	2. Fula [ ]	3. Wolof [ ]
4. Jola [ ]	5. Other, please specify: ✐	

6. Have you ever been to school?

1. Yes [ ]	2. No [ ]

If **'Yes'**, answer Questions 7 and 8. If **'No'** skip to Question 9

7. What type of school are you attending?

1. English [ ]	2. Arabic [ ]

8. What grade are you in currently?

1. Dropped out [ ]	2. Grade 1-6 [ ]	3. Grade 7-9 [ ]	4. Grade 10-12 [ ]

9. How many brothers and sisters do you have? Write 0 if you don't have any — Number: ✐

10. What type of family do you live in?

1. Nuclear family (father, mother, sister/s and brother/s)	[ ]
2. Extended family (father, mother, sister/s, brother/s, other family members)	[ ]
3. Single parent family (one parent: a mother or father)	[ ]
4. Step or blended family (two separate families coming together – for example a new husband or wife and children from previous marriages coming together)	[ ]
5. Grandparent family (parents of mother or father)	[ ]
6. Other family type not listed, please specify:	✐

11. Do you live in a rented home (e.g. apartment or house)

1. Yes [ ]	2. No [ ]

If **'Yes'** skip to Section 2. If '**No'** answer Question 12.

12. Who owns the home (e.g. the apartment or house) you currently live in? Tick **all** that apply if more than one owner.

A. My father	[ ]
B. My mother	[ ]
C. My grandparents	[ ]

D. Another family member		[ ]
E. A family friend		[ ]
F. Other family type not listed, please specify:	✐	

Section 2: Your health and social circumstances

13. Have you ever been married?

1. Yes [ ]	2. No [ ]

If **'Yes'** answer Questions 14 and 15. If **'No'** skip to Question 16.

14. Are you currently married?

1. Yes [ ]	2. No [ ]

15. How old were you when you got married?

Age (in years): ✐

16. How many children do you have? Write 0 if you don't have any

Number: ✐

17. Where do you get your source of drinking water? Tick **all** that apply if more than one source

A. Well (protected)		[ ]
B. Well (unprotected)		[ ]
C. Tap		[ ]
D. Travel to neighbouring village or town		[ ]
E. Other, please specify:	✐	

18. When did you last have malaria?

1. I currently have malaria	[ ]
2. Less than 3 months ago	[ ]
3. 3-6 months ago	[ ]
4. 7-12 months ago	[ ]
5. More than a year ago	[ ]
6. I have never had malaria	[ ]

19. When did you last receive deworming tablets?

1. Less than 3 months ago	[ ]
2. 3-6 months ago	[ ]
3. 7-12 months ago	[ ]
4. More than a year ago	[ ]
5. I have never taken deworming tablets	[ ]

20. What is your mother's occupation? Tick **all** that apply if more than one occupation.

1. Housewife	[ ]
2. Small trader	[ ]
3. Gardener	[ ]
4. Secretary	[ ]
5. Teacher	[ ]
6. Doctor	[ ]
7. Nurse	[ ]
8. Lawyer	[ ]
9. Cashier	[ ]
10. Journalist	[ ]
11. Farmer	[ ]
12. Accountant	[ ]
13. Domestic help	[ ]
14. Retired	[ ]
15. Not working	[ ]
16. My mother has died	[ ]
17. Other, please specify:	✐

21. What is your mother's highest level of education?

1. No formal education	[ ]
2. Primary/ secondary school	[ ]
3. Senior secondary school	[ ]
4. College	[ ]
5. University	[ ]

22. What is your father’s occupation? Tick **all** that apply if more than one occupation.

1. Tailor	[ ]
2. Teacher	[ ]
3. Carpenter	[ ]
4. Doctor	[ ]
5. Lawyer	[ ]
6. Small trader	[ ]
7. Merchant	[ ]
8. Gardener	[ ]
9. Nurse	[ ]
10. Farmer	[ ]
11. Journalist	[ ]
12. Driver	[ ]
13. Cashier	[ ]
14. Accountant	[ ]
15. Masoner	[ ]
16. Retired	[ ]
17. Not working	[ ]
18. My father has died	[ ]
19. Other, please specify:	✐

23. What is your father’s highest level of education?

1. No formal education	[ ]
2. Primary/ secondary school	[ ]
3. Senior secondary school	[ ]
4. College	[ ]
5. University	[ ]

Section 3: Community and household resources

24. What type of food markets do you have in your village? Tick **all** that apply

1. Standard build marketplace	[ ]
2. Open market (Lumo)	[ ]
3. Supermarket	[ ]
4. Shops	[ ]
5. Street food vendor	[ ]
6. Other, please specify:	✐

25. Do you or your parent/guardian shop for your household food items from your village?

1. Yes [ ]	2. No [ ]

If **'Yes'** answer Question 26. If **'No'** skip to Question 27.

26. Why do you or your parent/guardian travel to shop for your household food items? Tick **all** that apply

1. There is no market available in my village or town	[ ]
2. There are limited food choices	[ ]
3. It is cheaper	[ ]
4. Other, please specify:	✐

27. What type of healthcare services do you have in your village or town? Select all that apply? Tick **all** that apply

1. I don't have any in my community	[ ]
2. Hospital	[ ]
3. Health centre	[ ]
4. Clinic	[ ]
5. Pharmacy	[ ]
6. Village health worker	[ ]
7. Traditional midwife	[ ]
8. Other, please specify:	✐

28. Do you have electricity supply in your household?

1. Yes [ ]	2. No [ ]

If **'Yes'** answer Question 29. If **'No'** skip to Question 30.

29. What type of electricity supply do you have in your household? Tick **all** that apply

1. National Water and Electricity Company (NAWEC) supply	[ ]
2. Private installed solar	[ ]
3. Generator	[ ]
4. Other, please specify:	✐

30. Do you travel to attend school?

1. Yes, I travel to attend school	[ ]
2. No, I attend school in my village or town	[ ]
3. I am not attending school	[ ]

If **'Yes',** answer Question 31. If **'No'** or **'Not attending school'** skip to **Section 4**

31. Why do you travel to attend school?

1. There is no school available at my village/town	[ ]
2. The school/s cannot accommodate all my peers in the same grade	[ ]
3. Schools outside my village or town are of better quality	[ ]
4. I want to explore other schools in different communities	[ ]
5. My friends are attending that school	[ ]
6. I just like the school	[ ]
7. Other, please specify:	✐

Section 4: Sources of nutrition information

32. Where do you get information on nutrition-related health issues (this means how to eat healthily how health is affected by what we eat)? Tick **all** that apply.

1. I don't have access to nutrition information	[ ]
2. Family members	[ ]
3. Peers	[ ]
4. School	[ ]
5. Mass media	[ ]
6. Community members	[ ]
7. Health care practitioners	[ ]
8. I don't know	[ ]
9. Other, please specify:	✐

3. Parents Participant information Sheet

Participant Information Sheet
The 'Health-Secure Partnership':
Understanding rural adolescents' experiences and nutrition intervention needs

Your adolescent child is invited to participate in a research project that is being conducted by Leeds Beckett University (LBU) in collaboration with other universities in the UK, University of The Gambia, and the National Nutrition Agency (NaNA), The Gambia. The project is funded by the UK Medical Research Council.

The project aims to develop the first phase of a new nutrition intervention programme called '**The Health-Secure Partnership' (HSP)**. Before you decide that your child can participate, please read and discuss with them the details below, which provides you with information on why the research is being undertaken and what it will involve. Contact details are provided overleaf if you have any questions. We can also arrange a meeting for parents in early December 2024 at your Village Development Committee (VDC) Chairperson's compound.

Why is this research study being conducted?
In rural areas of The Gambia, among adolescents (young people aged 10-19 years) undernutrition (being underweight or short for your age) is very common. At the same time, more and more young people are experiencing overnutrition (being overweight or obese). Intervention programmes to address under or overnutrition among adolescents in rural Gambia have not been developed. The research project will be of interest to rural adolescents in The Gambia, their families, schools and communities.

Why have I been invited?
We would like to invite approximately 70 adolescents to participate within Mansakonko Local Government Area (LGA), The Gambia. In addition to talking to parents, teachers, and community health workers, we are interested in talking to adolescents to help us understand their experiences and needs so that we create HSP in ways that are best for them.

What will I be asked to do and how long will it take?
If you agree that your child can take part, they will be asked to complete a short questionnaire and then participate in a group discussion or one-to-one interview for about 1 hour. Overall, the research session should take no longer than two hours and will take place in your school or community during December 2024. Prior to their participation, you will be asked to read and sign a consent form to confirm and that you give consent to participation in the research session, and for the discussion or interview to be audio recorded to provide an accurate record of the conversations. We can read and explain the PIS and consent form to you in English or Mandinka, if you feel this would be helpful. When they arrive, we will outline to them what will happen during the research session and give them a chance to ask questions. The questions we will ask will be about them (e.g. your age, gender, and tribe), and their experiences and views on their health, behaviours or attitudes related to eating, activity, access to healthcare, and what they think will be essential to include in the HSP programme.

Do they have to take part?
No, they do not have to take part – participation in this research project is voluntary. Your child can stop participating in the research session at any time without giving a reason, with no harm to them, their education or their legal rights. If you or our child want to withdraw the information they have provided from the research project after the research session has taken place, please request this no later than 3rd February 2025. Contact the project researcher and this will be done using the *unique participant code* which will be assigned to your child's information (see below). After this time the analysis of the information will have begun and it will not be possible to separate out an individual's information.

Will taking part in this research project help me in any way?

You/ your child may not directly benefit from this research; however, their participation will improve the understanding of intervention needs for promoting healthy nutrition among Gambian adolescents. This will make a valuable contribution to designing the HSP programme.

Are there any potential safety risks in taking part?
There are no anticipated safety risks associated with completing the questionnaire. However, if any of the questions asked raises issues or concerns, we have provided ideas for further support below.

Will my/ my child's identity be kept confidential?
Any identifying information (such as you/ your child's name and contact details) will only be collected to organise the research session. Such information will be kept separately from what you tell us in the questionnaire, group discussion or interview. Therefore your identities will be kept confidential. **However, please note:** any information shared during the research sessions relating to anything that may be considered harmful to your child or others, will be disclosed to the appropriate authorities.

Leeds Beckett University processes data in line with the UK General Data Protection Regulation (GDPR). To understand how we collect, look after and share your data you should read the document accessed via this link: Confidentiality and Data Protection compliance. We can provide written copies if you do not have access to a computer.

What happens to the information provided?
The information provided during the research project is called **research data**. Your child's data will be assigned a unique participant code (e.g. P001), ensuring that their data will remain anonymous and confidential. This will include anonymous quotes from the discussions/ interviews. The research data from this project will be stored on a password protected computer system at LBU for use by the research team. All participant data will be kept for 10 years and then destroyed in December 2034.

Will the research be published?
The findings from the project will be used to produce a final report and publications such as academic papers. Conference presentations, social media posts, leaflets, videos and webpages will also be used to share our results with others – including the adolescents who take part and other members of the public. To help share the research for the benefit of society, data from the project which has been summarised (grouped together) and/or anonymised (no identifying information) will be placed in an online database at LBU (https://figshare.leedsbeckett.ac.uk). Anonymity and confidentiality will be maintained in everything produced from the research as it will not be possible to identify you/ your child in any way in these activities.

Who has reviewed this project?
This project has been reviewed and approved by LBU Local Research Ethics Coordinator (Ref: XXXX) and The Gambia Government/Medical Research Council (MRC) Joint Ethics committee (Ref: XXXX).

If you have questions about any aspect of this project, please phone the project researcher.

Project researcher:
Dr Haddy Jallow Badjan. Tel: +220 2961010 (or ask a family member, friend, or the VDC Chairperson to phone for you).

If you have a concern or complaint that you wish to discuss with someone independent from the researchers, please phone the Ethics Secretariat at the GG/MRCG JEC -Gambia Government/Medical Research Council Unit The Gambia Joint Ethics Committee (or ask a family member, friend, or the VDC Chairperson to phone for you).
Tel: +220 4495442

If participating in this project raises any issues or concerns you would like to discuss, you can access advice or support from your local school authorities, or visit a community health worker at Sandeng, Mansakonko Local Government, Region 3, The Gambia.

Thank you for reading this Participant Information Sheet

4. Adolescent consent form

<u>**Participant Consent Form**</u>

The 'Health-secure partnership': Understanding rural adolescents' experiences and nutrition intervention needs.

Please initial or thumbprint* the boxes below if you agree with the statements

1. I confirm that I have read and understood the information sheet (V1 03/09/24) for the above project. I have had the opportunity to consider the information, ask questions and have had these answered satisfactorily. ☐

OR

I have had the information explained to by study personnel in a language that I understand. I have had the opportunity to consider the information, ask questions and have these answered satisfactorily.

2. I can confirm that I am 18 years of age or older and I am providing consent for myself ☐

3. I understand that my participation is voluntary and that I am free to withdraw at any time, without giving any reason[1]. ☐

4. I understand that all information given is confidential and only for the project and that all data will be anonymised (names or other identifying information will be removed). ☐

5. I agree to take part in the project and to complete a questionnaire, take part in a focus group discussion (FGD) or to be interviewed. ☐

6. I agree to my FGD or interview being digitally recorded. I confirm information shared in the FGD by other participants will be respected and I will not disclose such information outside the group meeting. ☐

7. I consent to the processing of my personal information for the purposes explained to me. I understand that such information will be treated in accordance with the terms of the General Data Protection Regulation (GDPR) and the Data Protection Act 2018, and I have been given access to the Leeds Beckett University Research Participant Privacy Notice.

8. I agree that the summarised (grouped together) and/or anonymised (all personal identifying details removed) research data and results can be included/shared in a university repository (https://figshare.leedsbeckett.ac.uk) report, presentations, research papers, institutions, social media, radios, newspapers.

If you wish to take part in the project, please write and sign your name below

**Name of participant**	**Signature**	**Date**
✎	✎	✎

**Name of impartial witness**	**Signature of impartial witness***	**Date**
✎	✎	✎

[*Only required if the participant is unable to read or write.]

FOR COMPLETION BY THE RESEARCHER

I Dr Haddy Jallow Badjan, confirm that I have informed the above named about this research project. I have given them the Information Sheet. To the best of my knowledge, they have understood and have given free and informed consent to become a participant in the research project.

Signed .... Date ……………………………..............

[1]Participants can withdraw from the project at any point without giving a reason. However, to maintain anonymity and confidentiality we will keep your name separate from your data, and documents containing your information will be coded. For this reason, if you do complete a questionnaire, participate in a FGD or an interview but afterwards you wish to withdraw from the project, you can do this up to 3rd February 2025. After this time the analysis and summarising of the information will have begun, and it will not be possible to identify an individual's information.

5. Adolescent child assent form

Child Assent Form
The 'Health-secure partnership': Understanding rural adolescents' experiences and nutrition intervention needs.

My name is Haddy Jallow Badjan and I am a Gambian working at Leeds Beckett University, United Kingdom.

Why are we meeting with you?
I am here with other researchers because we are designing a programme of activities called an intervention. We use interventions to make sure that young people like yourself eat well and are healthy. We want to ask you what you think about food, health, and what would be good activities to have in the intervention.

What will happen to me in this project?
We will give you questions to answer but if you do not understand any words or questions, we will explain it to you. You will also take part in a discussion with me in a group or just you to talk about food, health, and the intervention.

Who will know that I am taking part in the project?
When we finished with this study, we will write a report about what we learned and share it with many people. Your name will not be included anywhere so no one will know that you were in the study unless you want to tell them.

Will I receive money if I take part in the project?
You will not be paid money for taking part, but we will give you some stationery as a thank you gift.

Do I have to take part?
You do not have to take part if you do not want to. If you decide to stop after we begin, that's okay too. You can stop at any time without explaining. You can ask for your details to be removed when we finish (or your parent can ask for you), but this must be asked for by 3rd February 2025. After this time all the information given to use will be combined and we will not be able to separate out the information that came from you.

Can I ask questions?
You can ask questions at any time. You can ask now. You can ask later. You can talk to us or you can talk to someone else at any time during the project. You will find my telephone number below if you want to talk.

If you are happy to take part in the project, please write your name and sign below.

If you wish to take part in the study, please write and sign your name or place your thumbprint* below

**Write your name or place your thumbprint* in the box** ☟	**Sign in the box** ☟	**Date**
✎	✎	✎

**Name of impartial witness**	**Signature of impartial witness***	**Date**
✐	✐	✐

[*Only required if the participant is unable to read or write.]

Researcher's signature……………………………………………………Date…………………

Contact details: Please phone Haddy Jallow Badjan on **+220 2961010** if you have any questions (or ask a family member, friend or the Village Development Committee Chairperson to phone for you).
